# Editorial for the Special Issue “Genetics and Genomics of Gastrointestinal Cancers: From Prevention to Treatment”

**DOI:** 10.3390/genes14091821

**Published:** 2023-09-20

**Authors:** Monica Marabelli, Lucio Bertario, Davide Serrano

**Affiliations:** Division of Cancer Prevention and Genetics, IEO, European Institute of Oncology IRCCS, 20141 Milan, Italy; monica.marabelli@ieo.it (M.M.); lucio.bertario@ieo.it (L.B.)

According to the latest estimate from GLOBOCAN 2020, approximately 18.1 million new cancer cases were diagnosed in 2020 around the world [1]. Gastrointestinal (GI) cancers are in the top five, accounting for 26% of the global cancer incidence and 35% of all cancer-related deaths [2]. Notably, it is estimated that at least 30% of cancers could be prevented through primary and secondary prevention programs [3]. However, despite the recent advances in early diagnosis, prevention, and therapies, the control and management of GI cancers remain challenging.

Cancer is a multifactorial disease, with genetic, epigenetic, and environmental factors having an impact on its occurrence and development. Carcinogenesis is a multistep process that involves the accumulation of genetic and epigenetic abnormalities, ultimately leading to malignancy [4]. A clear example of this progressive accumulation of multiple, clonally selected, genetic alterations was well described with the adenoma–carcinoma sequence for colorectal cancer (CRC) [5]. The genes involved in carcinogenesis often have a role in cell cycle regulation, DNA repair, apoptosis, and cell differentiation.

Besides “driver mutations” that cause the disease, cancer cells may further accumulate more abnormalities, leading to tumor heterogeneity [6]. These so-called “passenger” mutations are commonly acquired at the somatic level. Tumor-specific variants can have a prognostic or predictive effect and provide targets for new specific drugs. A recent study showed a very high frequency of these variants in CRC, with 10% of cases having molecular alterations already actionable for personalized treatment and another 10% showing changes associated with therapies already approved for other cancer types [7].

On the other hand, germline pathogenic variants are relatively rare and are responsible for hereditary cancer syndromes. These GI syndromes are characterized by a cancer risk up to 10 times higher than the general population [8]. This awareness is crucial not only for the patient, but also for the whole family, given the opportunity to identify high-risk relatives carrying a pathogenic mutation and, as a consequence, to promote personalized screening and preventive approaches.

Based on these considerations, we proposed a Special Issue with the title “Genetics and Genomics of Gastrointestinal Cancers: from Prevention to Treatment”. We present here an overview of the five original articles and two reviews included in this issue.

Calvello et al. [9] compared the genetic testing approach (single-gene vs. multi-gene panel testing) in a retrospective cohort of 54 gastric cancer (GC) patients. After a rigorous genetic risk assessment through pre-counseling, all participants underwent single-gene testing for one or more genes according to their personal or family history. A pathogenic variant was detected in nine (16.7%) patients. More specifically, seven out of fifty (14%) patients who underwent genetic testing for unknown mutations were carriers of a pathogenic variant in *CDH1*, *BRCA1*, *BRCA2*, and *MSH2* genes. A low rate (2%) of variants of unknown significance (VUSs) was identified. In addition, 37 out of 54 patients underwent multi-gene panel testing, including 29 cancer-related genes. Only two additional pathogenic variants emerged from this approach (in *ATM* and *RAD51D* genes), while VUSs were detected in 13 patients (35.1%). In conclusion, single-gene testing is still appropriate for the detection of pathogenic variants in patients fulfilling stringent selection criteria, especially if hereditary diffuse gastric cancer syndrome is suspected. A multi-gene panel approach could be preferred when unspecific phenotypes overlapping different cancer predisposition syndromes are present, but it has the disadvantage of leading to challenging results.

Whole Exome Sequencing is another approach for DNA analysis, useful in identifying genetic variants both at the germline and at the somatic level, and can represent a powerful tool to investigate hereditary predisposition to rare diseases. By using this technique, Nurgalieva and colleagues [10] analyzed genomic DNA from tumors and paired normal tissue samples in nine patients affected by gastric adenocarcinoma. They identified three pathogenic variants in two diffuse GC cases: a pathogenic variant in the *CDH1* gene in one patient and two pathogenic variants in the *VEGFA* and *FANCA* genes in a second patient. *CDH1* and *VEGFA* mutations were present only in tumor specimens, whereas the *FANCA* variant was found both in cancer and healthy tissues, indicating its germinal status. Notably, a germline pathogenic variant in *FANCA* has recently been reported also in CRC [11]. The association between *CDH1* genetic variants and diffuse GC is well established; according to the authors, *VEGFA* and *FANCA* could represent new possible candidate genes underlying GC development and deserve further investigation.

Besides genetic variants, epigenetic factors are known to be involved in cancer susceptibility and development. In particular, miRNAs can act either as oncogenes or tumor suppressor genes. Modesto’s group conducted a case–control study [12] with 301 GC patients and 145 controls by investigating a panel of 11 miRNA INDEL polymorphic variants. They found a significant correlation between five miRNA variants and GC development. In particular, germline variants in three miRNAs (miRNA4463, miRNA3945, and miRNA548H-4) were associated with an increased GC risk, whereas variants in miRNA920 and miRNA3652 provided a protective effect. An association with specific cancer features was also identified for miRNA4463_rs5877455, which appears to be associated with diffuse histotype, non-cardia localization, and early onset of the disease. The miRNA4463 variant, silencing the *PPP1R12B* tumor suppressor gene, could be a GC risk biomarker for early onset and poor prognosis.

Epigenetic and genetic changes are also involved in cancer progression, angiogenesis, and metastasis. In the paper by Zhang et al. [13], the role of angiopoietin-like proteins (ANGPTLs) was analyzed in colorectal cancer. ANGPTLs are a family of proteins structurally similar to angiopoietins. By means of several publicly available databases, the authors explored the expression profiles, prognostic values, genetic alterations, biological function, and immune infiltration correlation of seven ANGPTLs (ANGPTL1 to ANGPTL7) in CRC. Overall, the transcriptional level of these ANGPTLs was found to be lower in CRC tissues compared to normal tissues; however, protein expression was highly variable among the different ANGPTLs. Of relevance, the expression of ANGPTL4 was significantly positively correlated with the stage of CRC, and high levels of ANGPTL1, -2, and -6 were associated with a poorer cancer prognosis. More specifically, in vitro models showed that ANGPTL4 could promote proliferation and migration of CRC cells. Further investigation revealed that ANGPTLs participate in signal transduction and regulation of transcription and are involved in different cancer-related pathways. The authors hypothesized that ANGPTLs could be potential drug targets and, in particular, in silico analyses allowed them to identify four specific small molecules potentially able to bind ANGPTL4. Additional cellular and molecular analyses and in vivo animal experiments are required to better clarify the prognostic value and potential therapeutic applications of ANGPTLs in CRC.

An uncommon and very aggressive form of CRC, rhabdoid colorectal tumors (RCTs), has been deeply investigated by Remo and colleagues [14]. They examined 21 patients with RCTs using immunohistochemistry and next-generation sequencing. Rhabdoid tumors are usually located in the kidney and arise in childhood. Although pediatric tumors are often characterized by genetic alterations in *SMARCB1*, the authors found that SMARCB1 expression was present in the great majority of RCT cases (85%), supporting the idea that this chromatin remodeling gene may not be so relevant in adults. Loss of nuclear expression of MMR proteins was detected in 13 patients (62%). The epithelial markers CDX2 and CK20 were negative in the majority of patients, reaching 80% in the MMR-deficient subgroup. Of relevance, activating mutations in the MAPK pathway were detected in more than 70% of the lesions, mainly *BRAF* V600E. Moreover, this study demonstrated that primary cilia are involved in the acquisition of rhabdoid features. Ciliogenic markers such as Ciliary Rootlet Coiled-Coil (CROCC) and γ-tubulin were globally altered in these tumors and were found to colocalize in large cilia on cancer tissues and not in normal controls. Altogether, their findings suggest that the aggressiveness of RCTs may be due to alterations in primary cilia and MAPK pathway activation; accordingly, they could represent new therapeutic targets.

Concerning CRC treatment, microsatellite instability (MSI) has taken an increasingly relevant role. MSI is found in approximately 15% of all CRCs; 3% are diagnosed in patients with Lynch syndrome, while the remaining 12% are sporadic cases. Irrespective of hereditary or sporadic etiology, Greco and colleagues [15] reviewed the role of MSI as a biomarker in the diagnosis, prognosis, and treatment of CRC, with a focus on immune interactions within the tumor microenvironment and their therapeutic applications. The MSI phenotype arises as a consequence of a defective mismatch repair (dMMR) system, which leads to the accumulation of genetic alterations. Due to this high mutational burden, MSI CRC cells express many surface neoantigens that trigger an important immune response. Accordingly, CRCs with the MSI phenotype have a greater intra-tumor infiltration of T lymphocytes and exhibit a tumoral microenvironment that reduces the metastatic potential. Indeed, lymphocyte infiltration may be related to the observed better prognosis in early-stage cancers. Neoplastic cells with dMMR can eventually overexpress several immune checkpoint proteins, including PD-1 and PD-L1, to reduce the efficiency of the cytotoxic immune response. These molecules can be targeted through immunotherapy agents, such as Pembrolizumab or Nivolumab, leading to overcome this blockade and restore the immune response toward cancer cells. Accordingly, in the presence of late-stage CRC, MSI can be a predictive marker for responsiveness to immunotherapy targeting immune checkpoints blockade.

Celiac disease (CD) may be associated with an increased risk of several malignancies; in particular, it increases the risk of cancer within the gastrointestinal tract up to 60%. Ivanova’s group presented a review [16] on genetic and epigenetic factors possibly explaining the relation between CD and cancer. After a comprehensive overview of genomics, epigenomics, and transcriptomics data on CD, the authors analyzed common cancer hallmarks in celiac patients. Alterations in several genes, including *ELMO1*, *ATXN2*, *ITGA4*, and *PSMA8*, have been implicated in both CD and esophagogastric cancer pathogenesis. *APC* promoter hypermethylation has been found in 73% of CD-associated small intestine malignancies. Furthermore, small bowel carcinomas arising from celiac patients are often characterized by MSI with *MLH1* promoter hypermethylation. The association between CD and CRC is less striking and still debated. A long non-coding RNA, IQCJ-SCHIP1-AS1, could be involved in pathways shared by the two diseases. Validation of these biomarkers in celiac patients may help to identify at-risk individuals and lead to early disease detection. Expanding our knowledge of the biological interplay between CD and cancer will have significant implications in terms of clinical management and screening protocols.

The articles of this Special Issue cover a very wide range of topics, from the genetic testing approach to prognostic markers, from molecular pathways of carcinogenesis to the identification of possible targets for new therapeutic approaches. Some aspects may be more practical and already applicable; some others will require a longer validation process. Genetics and epigenetics studies are increasingly crucial in the era of precision medicine and will hopefully lead to advances in the diagnosis, prevention, and treatment of GI cancers.
